# A Platform and Multisided Market for Translational, Software-Defined Medical Procedures in the Operating Room (OP 4.1): Proof-of-Concept Study

**DOI:** 10.2196/27743

**Published:** 2022-01-20

**Authors:** Magdalena Görtz, Michael Byczkowski, Mathias Rath, Viktoria Schütz, Philipp Reimold, Claudia Gasch, Tobias Simpfendörfer, Keno März, Alexander Seitel, Marco Nolden, Tobias Ross, Diana Mindroc-Filimon, Dominik Michael, Jasmin Metzger, Sinan Onogur, Stefanie Speidel, Lars Mündermann, Johannes Fallert, Michael Müller, Magnus von Knebel Doeberitz, Dogu Teber, Peter Seitz, Lena Maier-Hein, Stefan Duensing, Markus Hohenfellner

**Affiliations:** 1 Department of Urology Heidelberg University Hospital Heidelberg Germany; 2 SAP SE Walldorf Germany; 3 German Cancer Research Center Heidelberg Germany; 4 National Center for Tumor Diseases Dresden Germany; 5 KARL STORZ SE & Co. KG Tuttlingen Germany; 6 mbits imaging GmbH Heidelberg Germany; 7 Department of Applied Tumor Biology Institute of Pathology Heidelberg University Hospital Heidelberg Germany; 8 Department of Urology Städtisches Klinikum Karlsruhe Karlsruhe Germany; 9 Siemens Healthineers AG Forchheim Germany; 10 Section of Molecular Urooncology Department of Urology University of Heidelberg School of Medicine Heidelberg Germany

**Keywords:** cloud-based platform, data, eHealth, Internet of Medical Things, IoT, medical apps, multisided market, perioperative medicine, software-defined healthcare, translational research

## Abstract

**Background:**

Although digital and data-based technologies are widespread in various industries in the context of Industry 4.0, the use of smart connected devices in health care is still in its infancy. Innovative solutions for the medical environment are affected by difficult access to medical device data and high barriers to market entry because of proprietary systems.

**Objective:**

In the proof-of-concept project OP 4.1, we show the business viability of connecting and augmenting medical devices and data through software add-ons by giving companies a technical and commercial platform for the development, implementation, distribution, and billing of innovative software solutions.

**Methods:**

The creation of a central platform prototype requires the collaboration of several independent market contenders, including medical users, software developers, medical device manufacturers, and platform providers. A dedicated consortium of clinical and scientific partners as well as industry partners was set up.

**Results:**

We demonstrate the successful development of the prototype of a user-centric, open, and extensible platform for the intelligent support of processes starting with the operating room. By connecting heterogeneous data sources and medical devices from different manufacturers and making them accessible for software developers and medical users, the cloud-based platform OP 4.1 enables the augmentation of medical devices and procedures through software-based solutions. The platform also allows for the demand-oriented billing of apps and medical devices, thus permitting software-based solutions to fast-track their economic development and become commercially successful.

**Conclusions:**

The technology and business platform OP 4.1 creates a multisided market for the successful development, implementation, distribution, and billing of new software solutions in the operating room and in the health care sector in general. Consequently, software-based medical innovation can be translated into clinical routine quickly, efficiently, and cost-effectively, optimizing the treatment of patients through smartly assisted procedures.

## Introduction

### Background

Innovation and growth in the health care sector could be significantly improved by supporting the rapid translation of software-based medical research and its results into clinical routine, increasing patient outcomes at scale. Technological advances such as the improved visualization of target structures during surgery by means of augmented reality [1,2] promise to further enhance the outcomes of surgery for the greater benefit of the patient. However, the development and translation of navigated, software-based innovations into commercial solutions are affected by two main challenges: because of proprietary systems, access to medical devices and their data is difficult [3], and there are high barriers to the transfer of research results into clinical practice, particularly a successful market entry [4]. Compared with Industry 4.0, the health care sector so far has not prepared for similar developments. As an example, medical devices commonly represent highly specialized but unconnected stand-alone solutions, optimized for their task but with limited flexibility and extensibility [5]. Start-ups offer high promises of disruptive innovation in the health care sector because they are highly flexible and make use of new technologies [6]. However, a fast and efficient go-to-market is especially difficult for small companies, start-ups, and spin-offs of research institutes. High barriers to market entry result in delays or failure to bring innovative solutions into clinical routine where their benefits could help larger numbers of patients [7].

The proof-of-concept project OP 4.1 addresses this issue by providing software-based solutions with connection to devices and data, consequently supporting their translation into clinical routine. With OP 4.1, we demonstrate for the first time an open and extensible platform prototype that is not only *open to join*, creating an open ecosystem, but also allows comprehensive connectivity and augmentation of the physical capabilities of medical devices through software-based add-ons, enabling the fast implementation of new solutions in the operating room. The open and extensible design of the platform offers developers well-established and standardized interfaces for stakeholders to connect their apps easier and more efficiently compared with closed systems with proprietary interfaces. The use of open, nonproprietary interfaces in the OP 4.1 platform eases interoperability and data exchange among stakeholders and is important for widespread adoption. In addition, the platform is not limited to a fixed set of interfaces; it can be extended to provide future standards or individual needs.

### Software-Defined Healthcare

We introduce the term *software-defined healthcare* in accordance with the definition of *software-defined vehicles*, highlighting that software is becoming the driving factor of innovation and a key value generator, whereas hardware is becoming more and more standardized and eventually commoditized, mostly acting as a base to build differentiating capabilities through software on top [8]. The term builds on the notion of *software-defined systems*, where the software components are segregated from the underlying hardware by means of different abstraction layers [9]. As can be observed in various industries (eg, in the automotive industry), it is easier to update software without having to change hardware, and this also has great potential for continuous innovation and further differentiation. An early example from the automotive sector is the intercompany collaboration on car platforms as well as the use of digital (entertainment) systems as a means of differentiation in current marketing by vendors. In this paper, we focus on software-defined medical procedures in the operating room and how they can be enabled technically and commercially by introducing an underlying platform with a multisided market. We proofed the efficacy of the proposed solution by successfully developing 4 apps for use in the operating room (*augmented reality app*, *live perfusion measurement app*, *precision puncture app*, and *mobile information service app*). Through the introduction of the business and technology platform OP 4.1, we effectively created a multisided market for medical device services, allowing for fast commercialization of software-based research solutions in the operating room and in the health care sector in general.

## Methods

### Stakeholders Required to Design a Multisided Market

**Figure 1:**
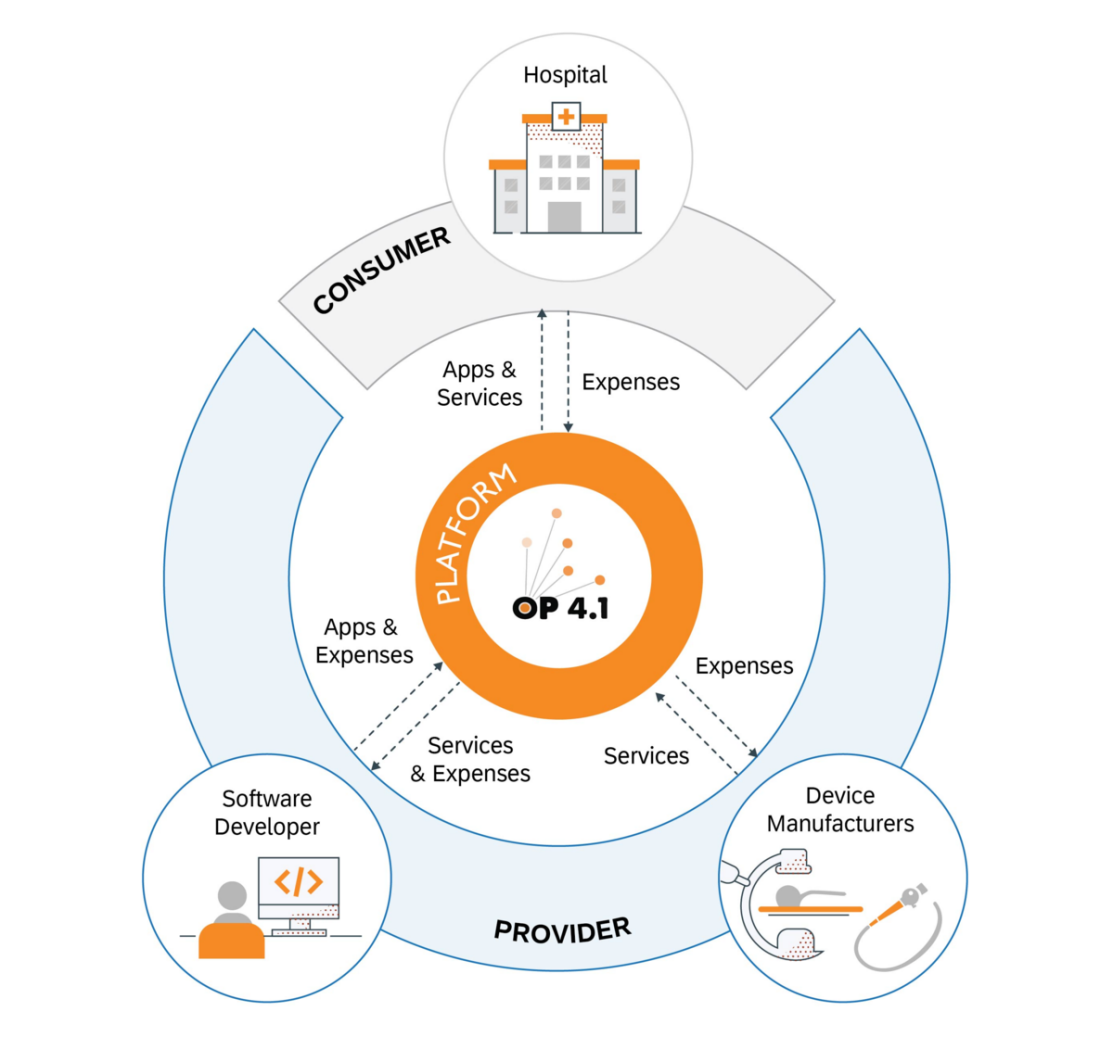
OP 4.1 platform business model. The OP 4.1 platform approach creates value for all parties involved by facilitating exchanges among several independent market contenders. Through the platform, consumers (eg, hospitals) and providers (eg, software developers and device manufacturers) interact with each other, with providers interacting among themselves as well. Hospitals pay software developers and device manufacturers for apps and services, software developers consuming device services pay device manufacturers for consuming these services, and software developers charge device manufacturers for software solutions such as predictive maintenance.

The creation of a central platform prototype requires the active collaboration of several independent market contenders. These consist of *consumers*, *providers*, and platform *suppliers* (Figure 1).

The primary *consumers* on the OP 4.1 platform are medical staff, the hospital administration, and the information technology (IT) department. The *consumers* consume services made available by the *providers* on the platform. The platform’s various user spaces must be designed around the requirements of the *consumer* and the medical staff, considering the process of buying, testing, and rating the solutions. In this proof-of-concept study, the *consumer* was the Department of Urology at Heidelberg University Hospital, which initiated the project OP 4.1.

*Providers* in the context of the OP 4.1 platform are primarily software developers and their companies (eg, start-up or research organizations with their potential spin-offs) and device manufacturers. A software developer in the OP 4.1 consortium was the Deutsches Krebsforschungszentrum (DKFZ; German Cancer Research Center). The role of the DKFZ in the project OP 4.1 was to provide a starting set of services to extend the platform’s capabilities, to develop basic infrastructural components for developers to extend the OP 4.1 platform, and to use the OP 4.1 platform to develop apps. The goal was to exemplify how to implement existing research projects of the DKFZ on the OP 4.1 platform in the form of apps [10-12]. The set of research projects of the DKFZ comprised three apps: 1 for preoperative planning and intraoperative assistance of laparoscopic kidney tumor resections (*augmented reality app*) [10], 1 for live perfusion monitoring based on multispectral imaging data (*live perfusion measurement app*) [11], and 1 for a marker-less navigation concept for high-precision needle punctures (*precision puncture app*) [12]. The goal of the fourth app, *mobile information service*, was to disseminate information about the current state and progress of surgeries to mobile devices connected to the OP 4.1 platform. This contribution to OP 4.1 was provided by the start-up company mbits imaging GmbH, the other software developer in the consortium.

As outlined in the previous paragraph, medical device manufacturers are also *providers* on the OP 4.1 platform. They could connect their devices to the platform to supply data (eg, usage data) and provide their device-generated data to software developers according to a price stipulated by them. In the OP 4.1 project, this role was undertaken by the medical device manufacturer KARL STORZ SE & Co. KG, whose contribution to OP 4.1 was to supply a gateway for the standardized acquisition of surgical data streams in the operating room and to facilitate the interface between clinical devices and the cloud-based integration platform. Another medical device manufacturer who joined the OP 4.1 project was Siemens Healthineers AG to enable the *augmented reality* app with intraoperative 3D imaging.

The platform connects *providers* and *consumers* and generates network effects. For the success of the platform, a neutral platform owner, one that is neither *consumer* nor *provider*, is advisable to attract as many market contenders as possible without them having to fear direct competition. This setup is particularly relevant for the medical device manufacturers’ side to be able to provide as much choice to the consumers as possible. With SAP SE, a partner for software applications, user-centric design, and, with the SAP Cloud Platform, service-oriented commercial platform solutions, strengthened the OP 4.1 consortium. The goal of SAP SE as the platform *supplier* was also to create a holistic business platform model for this specific scenario based on earlier conceptual work by Cigaina [13].

### Approach Required to Implement a Platform for a Multisided Market

In the OP 4.1 consortium, an agile design and development process was used to ensure that the requirements for the technical platform and apps include the needs of all envisioned user types, for example, physicians and developers. The project culminated in the final prototype that was presented at the conclusion of the project in January 2020 at a demonstrator event for the German Federal Ministry for Economic Affairs and Energy, the project governance body Deutsche Gesellschaft für Luft- und Raumfahrt (German Aerospace Center), the general public, and the media.

### Technology Architecture of the OP 4.1 Platform to Establish a Multisided Market

**Figure 2:**
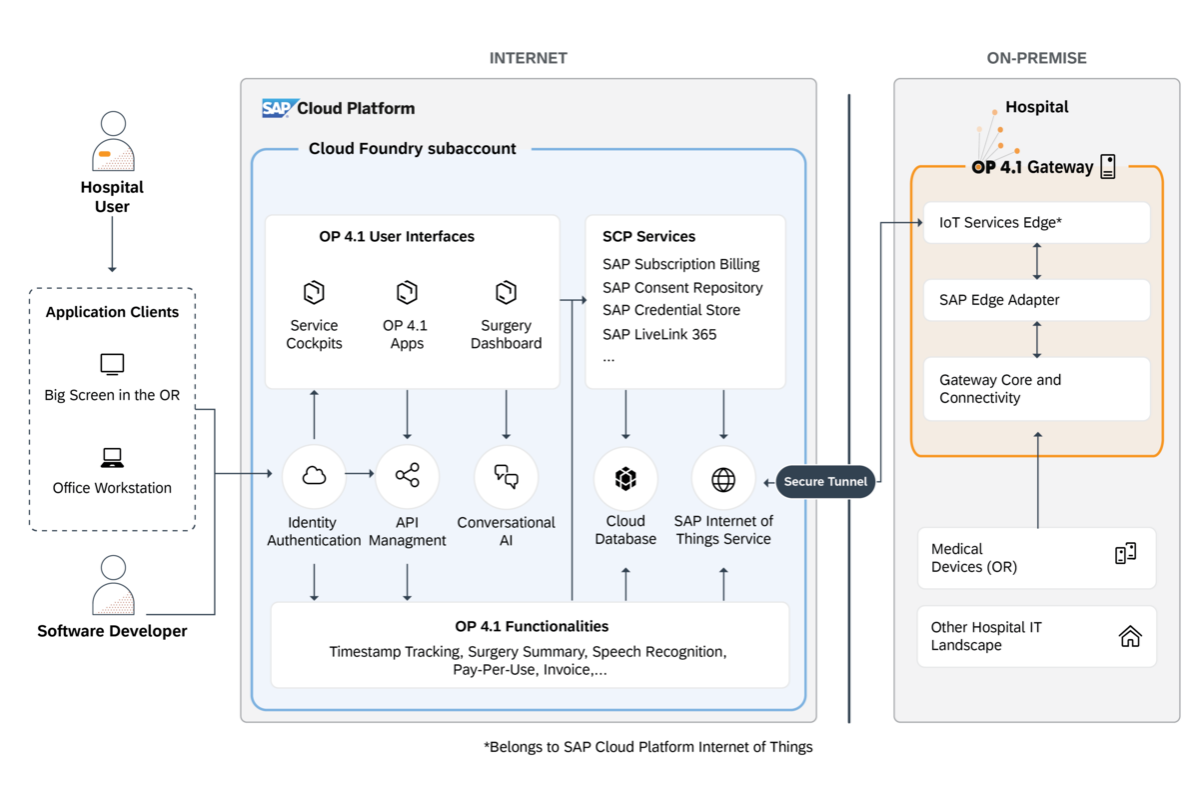
OP 4.1 architecture. The OP 4.1 platform consists of two instances: one that is cloud-based and built on SCP and one that is on the premises at the hospital, consisting of the OP 4.1 gateway, connected medical devices, and relevant parts of the hospital’s IT landscape. On the cloud side, a Cloud Foundry subaccount holds OP 4.1 core components such as identity authentication, API management, cloud database, and IoT services, as well as conversational AI, complemented by functionalities related to ORs, for example, timestamp tracking, surgery summary, speech recognition, pay per use, and invoice. These OP 4.1 core components leverage standard SCP services such as SAP Subscription Billing, SAP Consent Repository, SAP Credential Store, and SAP LiveLink 365 supporting various billing scenarios, documenting patient consent, and managing user authorization. Access to the functionalities and services is provided by the OP 4.1 user interfaces, primarily the service cockpits, the provided OP 4.1 apps, and the surgery dashboard. These can be accessed by hospital user and software developers through screens in the OR and office workstations. Direct access to the platform for software developers is also provided. On the on-premises side, we developed the OP 4.1 gateway for the standardized acquisition of surgical data streams in the OR and for the facilitation of the interface between medical devices and the cloud-based OP 4.1 integration platform through a secure tunnel. The SAP Edge Adapter and the IoT Services Edge together with the Gateway Core and Connectivity implement the required data models to publish the device data for SCP. The IoT Services Edge enables decentralized data processing at the edge of the network. This affords the possibility to process data and services locally. Through the IoT Services Edge, processed or aggregated data can be sent from the OP 4.1 gateway to the cloud. The SAP Edge Adapter we developed for this project is a Kafka-to–MQ Telemetry Transport adapter and connects the Gateway Core to the IoT Services Edge. The Gateway Core contains, among others, a STORZ Communication Bus client, an Online Certificate Status Protocol client, and the Apache Kafka component. AI: artificial intelligence; API: application programming interface; IoT: internet of things; IT: information technology; OR: operating room; SCP: SAP Cloud Platform.

The OP 4.1 platform consists of two instances: 1 that is cloud-based and built on SAP Cloud Platform [14] and 1 that is on the premises at the hospital, consisting of the OP 4.1 gateway, connected medical devices, and relevant parts of the hospital’s IT landscape (Figure 2).

On the cloud side, a Cloud Foundry (Cloud Foundry Foundation) subaccount [15] holds the OP 4.1 core components such as identity authentication, application programming interface (API) management, cloud database, and internet of things (IoT) services, as well as conversational artificial intelligence (AI), complemented by functionalities related to operating rooms, for example, timestamp tracking, surgery summary, speech recognition, pay per use, and invoice. These OP 4.1 core components leverage standard SAP Cloud Platform services such as SAP Subscription Billing [16], SAP Consent Repository [17], SAP Credential Store [18], and SAP LiveLink 365 [19]. Access to the functionalities and services is provided by the OP 4.1 user interfaces, primarily the service cockpits, the provided OP 4.1 apps, and the surgery dashboard. These can be accessed by the hospital user and software developers through their common interaction devices such as screens in the operating room for the surgeons and office workstations for the developers.

On the on-premises side, we developed the OP 4.1 gateway for the standardized acquisition of surgical data streams in the operating room and for the facilitation of the interface between clinical devices and the cloud-based OP 4.1 integration platform through a secure tunnel. The OP 4.1 gateway provides *connectivity* (eg, data import and preprocessing) by transferring relevant data from devices and existing data sources using interfaces, *interoperability* (eg, adjustment of data types and formats) by definition of data formats, standardization, and dedicated selection of data for analysis, as well as *distribution*, by provisioning data to further systems.

In the OP 4.1 project, a number of interfaces were implemented to connect various medical devices and IT systems. These include, for example, the STORZ Communication Bus [20] as well as emerging standards for the interoperability of medical systems, such as IEEE 11073 Service-Oriented Device Connectivity [21,22], HL7/Fast Healthcare Interoperability Resources [23,24], and IHE Patient Care Device [25]; video capture; and a digital imaging and communications in medicine [26] node. An Apache Kafka (Apache Software Foundation) [27] cluster was integrated to convert the incoming data streams where necessary and to provide real-time data feeds to the cloud instance. The interfaces also allow the connecting of existing parts of the hospital’s IT landscape, such as the picture archiving and communication system or hospital information system (HIS), to the OP 4.1 platform, augmenting their capabilities and enabling a holistic overview of all related information. Thereby, the existing data from these systems are not copied to the OP 4.1 platform; rather, related metadata are exposed to enable system access when required.

The generic OP 4.1 platform can be regarded as having interoperability at the structural level (level 2) of the Health Information Management and Systems Society categories [28]. To reach higher levels of interoperability, there are 2 approaches available: on the one hand, platform extensions that supply support for additional interface standards; on the other hand, content extensions that provide semantic layers. The latter, in particular, represents a commercial opportunity for third parties implementing Systemized Nomenclature of Medicine [29] or related standards. As the platform is open and extensible, all conceivable standards that are not yet inherently delivered with a productive instance of the platform can be provided by third parties. Software developers who would like to leverage a semantic layer can build proprietary add-ons to integrate data into a semantic model. The open and extensible OP 4.1 platform also allows software developers to create and make available their own semantic layer to other software developers so that it can be used not only for individual apps, but it is also established as a central element, extending the platform. Given such extensions, it is envisionable to be able to reach higher levels of interoperability, such as semantic level 3 and beyond.

In an active setting, the use of a medical device would trigger an OP 4.1 gateway usage event, which is later processed by SAP IoT Services Edge [30] and provisioned to the cloud-based event handler. The event handler in turn creates a usage record with SAP Subscription Billing. Once triggered, the invoicing functionality processes all available usage records and generates a consolidated invoice according to the respective billing rules.

### Data Protection and Security Concept Required to Apply a Platform in Clinical Routine

To ensure broad acceptance of the OP 4.1 platform, we created a data protection and security concept. When used in a real-life scenario, OP 4.1 requires conformity with relevant data protection laws when handling patient data [31]. Since May 2018, the General Data Protection Regulation 2016/679, a regulation in European Union law on data protection and privacy, has been the binding directive in the European Union and the European Economic Area [32]. On the basis of this data protection regulation, we generated an OP 4.1 data protection checklist for software developers. Data processing systems must be protected against unauthorized use, and only authorized persons should have access to the data for which they have been granted specific access rights. This was technically implemented using the Open Authorization 2.0 authentication protocol [33] and several other security technologies and capabilities provided by the underlying SAP Cloud Platform such as the SAP Authorization and Trust Management Service [34], the SAP Cloud Identity Services [35], and the SAP Trust Center [36], which are set up in a fenced network. The OP 4.1 platform prototype runs in a shared environment where the data are isolated from each other and the traffic is controlled by firewalls. Administrative access is performed through terminal services that require strong authentication. All communication channels are protected with the transport layer security protocol. Proper user authorizations are also required and respected: for example, when an HIS is connected to the platform, the platform is able to allow its users access to the HIS through the dashboard. However, the user needs to be authorized against the platform, and as in well-established single–sign-on scenarios, the platform passes on the user request to the HIS for further processing. There, the user request is checked to determine if the user is authorized to access the requested parts of the target system (eg, the HIS) so that access can be granted. Thus, users do not have automatic access to all data or systems through the platform; only users who have permission to use the respective parts of the connected systems (eg, the HIS) can access it through the platform.

We established that the integration platform enables apps to log access to data, including but not limited to username and access date. All processes need to include an option for correction, anonymization, and deletion. In real-world use, affected patients must agree that their health-related data are collected, processed, and used on the cloud-based platform and made available to its respective parts.

## Results

### The OP 4.1 Platform as a Basis for the Implementation of Translational Apps in Clinical Routine

**Figure 3:**
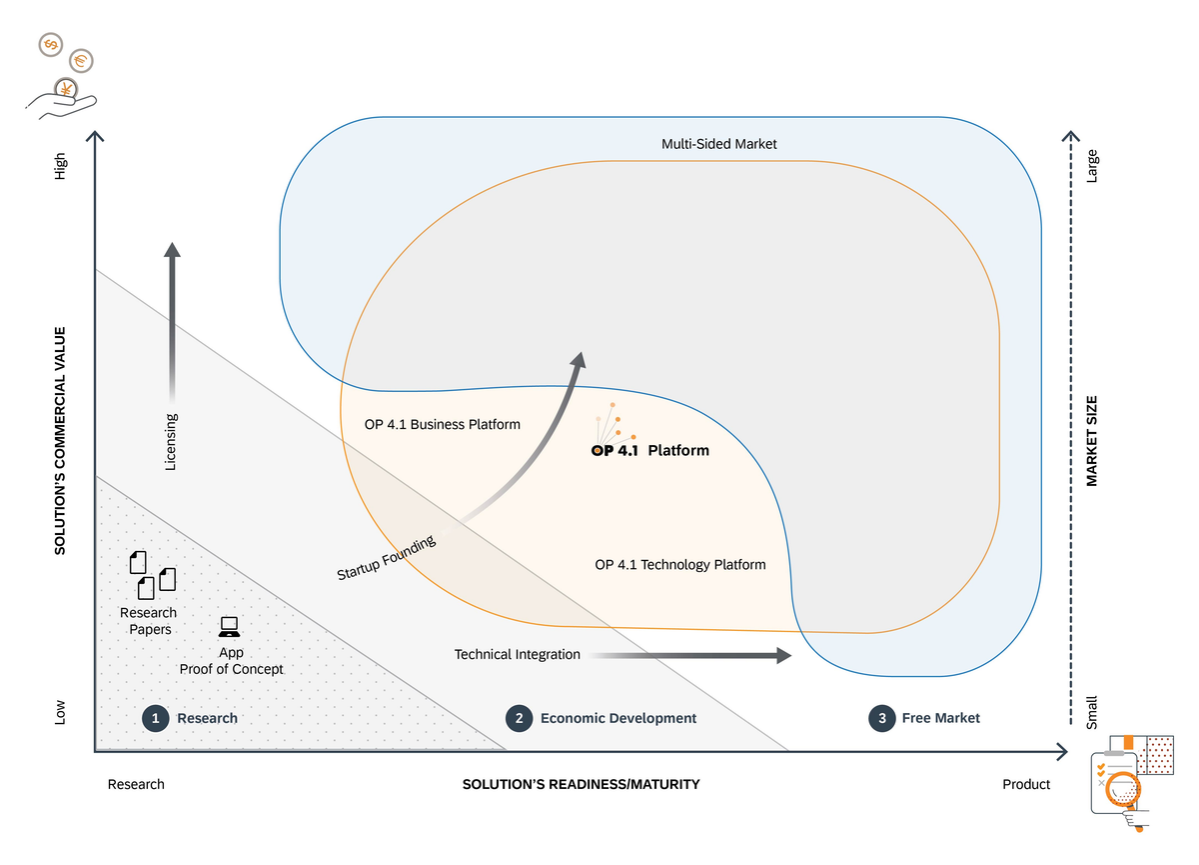
OP 4.1 approach. Most research organizations are funded through research grants; their work results in research papers and, in the area of software-defined healthcare, apps as proofs of concept. The readiness and maturity of such solutions is often low, and there is very limited commercial value to capture because of their nature as proofs of concept. To overcome these challenges, develop economically, and generate revenue outside of research grants, there are 3 different dimensions to consider: first, protect and license the innovation, which increases its commercial value; second, increase the solution’s readiness through further technical integration, effectively providing apps that come closer to a more mature product state; and third, found a start-up to commercialize the innovation or invention. By choosing the third option, the start-up needs to not only show increasing commercial value to investors but also increase the solution’s readiness beyond the initial proof of concept. Eventually, there needs to be a market, especially for such purely software-based solutions, which, because they mostly require medical devices to operate with, is a multisided market with different types of market contenders interacting on a platform. This is where the OP 4.1 platform comes into play. On the side of the solution’s readiness, the technology platform side of OP 4.1 provides an environment to efficiently execute the solution, supporting the entire development and deployment cycle. On the side of commercial value, the focus is on OP 4.1 as a business platform: by being able to not only bring a solution to market but also charge for it, commercialization becomes possible, and the access to the market created by the OP 4.1 platform enlarges the addressable market of the start-up’s solution, effectively increasing its commercial value. As a result, OP 4.1 creates a multisided market by providing a technical and business platform to help research-based solutions to fast-track their economic development and become commercially successful.

The ultimate goal of OP 4.1 is to create a multisided market by providing a technical and business platform to help research-based solutions to fast-track their economic development and become commercially successful (Figure 3). To support the effective translation of software-based solutions into clinical routine, we developed the OP 4.1 platform prototype. As described previously, the OP 4.1 platform has the capability to provide standardized and open interfaces to devices and data sources, integrate heterogeneous data, and provide central services (eg, data modeling and processing, user administration, and access management) as well as development and commercial support, all combined with a user-centric design (Figure 2).

The platform provides clinical process data (eg, image and video data, vital signs, and device data) and an expandable selection of platform services (APIs) to its users. Moreover, platform apps can be built using the software development kit of the OP 4.1 platform with predefined design and interaction concepts, the documentation of previous apps, and the OP 4.1 API Hub. The API Hub serves as the central instance for searching for APIs to use within apps on the OP 4.1 platform prototype. The API Hub itself is a web portal application, documenting use and prices and allowing APIs to be evaluated, downloaded, and tested. The APIs, which are provided as services to all apps, are functions that are technically validated by the platform provider, medical device manufacturer, or software developer through the certification of the API-providing device or data source, and they extend the core platform capabilities. The APIs are provided on the platform to developers for creating software or for interacting with systems. Thus, the APIs often serve common functionalities to reduce the development effort for software developers and to enable easy access to device and other data, supporting intuitive development on the platform. The concept allows third-party companies to develop their software solutions directly on the development instance of the OP 4.1 platform, testing them against digital devices offered by medical device manufacturers, running quality checks on the platform’s quality instance, ideally having their solutions’ technical interfaces automatically precertified, and eventually deploying them into the hospital’s own platform space. It needs to be noted that although the OP 4.1 platform can provide a certification of the technical interoperability of apps and medical devices at an interface level to ensure seamless integration with the platform, medical certifications for each app need to be fulfilled additionally by the respective software developer. Medical products, including apps, can be certified modularly and independently of each other, and their sensible combination is then also permissible. In the case of OP 4.1, not only the respective apps and the connected medical devices, but also the OP 4.1 gateway as a specific medical device must be certified.

However, the OP 4.1 platform makes all usage data available for authorized users and provides a unified environment for developing, testing, deployment, and runtime of apps. Thus, by providing the relevant data and direct information to support medical certification in a standardized and repeatable manner, the translation of software solutions into clinical routine can be fast-tracked through the OP 4.1 platform compared with the time-consuming process of certification of solutions for different stand-alone environments. In addition, the platform’s standard environment with modular functionality means that certification can take place more efficiently because the basic underlying functionality remains the same across apps and its descriptions and reviews can be easily reused for new use cases on top of the OP 4.1 platform.

Although many apps will be cloud-only, thus mostly soft real-time, there are specific use cases when solutions from OP 4.1 are not only deployed in the customer’s cloud space, but will also be partially delivered on special appliances at runtime because they might require highly hardware-dependent functionality, including but not limited to hard real-time (eg, high-definition 3D videos and graphics processing unit arrays for augmented reality or deep learning algorithms). In this case, the platform still controls all related metadata and data storage locations to perform the platform’s services such as authorization, updates, connection, charging, billing, and invoicing.

To our knowledge, the OP 4.1 platform is the first holistic cloud-based platform that supports developers in designing, coding, testing, deploying, maintaining, and commercializing their innovative solutions for the operating room.

### The OP 4.1 Gateway to Connect the Medical World With the IT World

To apply the OP 4.1 platform in the operating room, it needs to be connected to the various medical devices and appliances. As a well-defined link among these independent medical devices with IT solutions, including a cloud-based integration platform such as OP 4.1, we introduced the OP 4.1 gateway (Figure 2). Different interfaces were implemented to connect various medical devices, data sources, and IT systems (as described in the *Methods* section). The gateway locally aggregates the intraoperatively acquired multimodal data, preprocesses them, and makes them available to the central platform layer. The gateway also ensures proper import, standardization, and distribution of data, while promoting data connectivity and interoperability. Data persistence is achieved through connected systems and data lakes such as the picture archiving and communication system.

### The OP 4.1 Dashboard and Apps to Prove the Translational Capabilities in the Clinic

**Figure 4:**
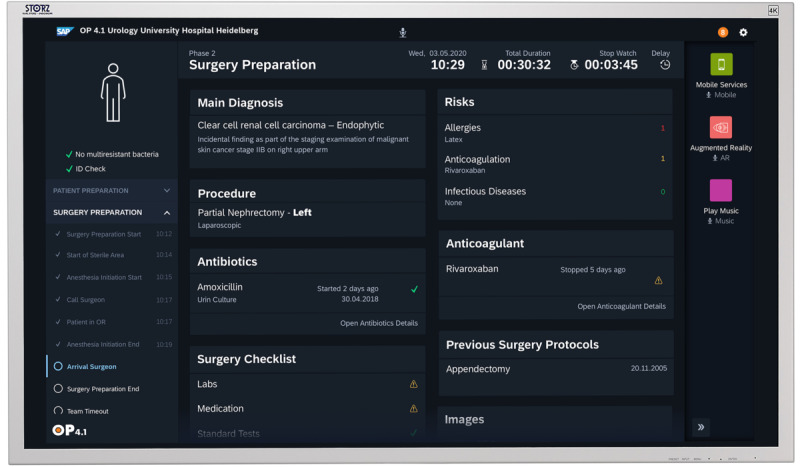
OP 4.1 user-centric dashboard with access to medical data and apps. The context-sensitive dashboard enables users to control the entire OP 4.1 platform before, during, and after surgery. It also provides seamless access to information from external sources such as the hospital information system and allows the starting of preselected apps.

**Figure 5:**
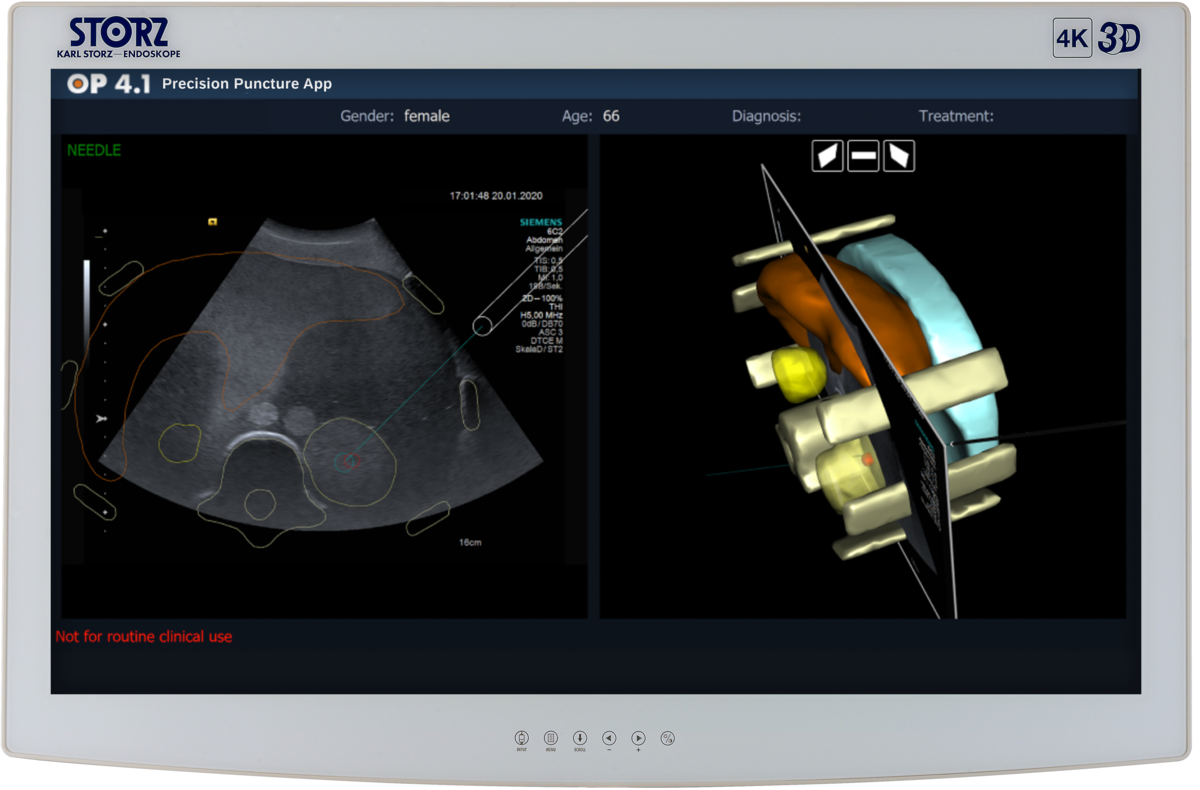
OP 4.1 precision puncture app. By combining live ultrasound images with segmented computed tomography images from connected systems through the central OP 4.1 platform, this app enables a safe and fast percutaneous puncture of target structures, for example, in the kidney. Left: ultrasound image with the projection of the needle and the segmented structures from the computed tomography images. Right: 3D scene, including the ultrasound plane and the segmentations, made possible by multimodal image fusion.

To demonstrate the efficacy of the OP 4.1 platform, we created a platform dashboard and 4 platform apps. The dashboard is a proof of concept for using platform information and for making the integrated platform visible to the end user. With the context-sensitive dashboard based on a user-centric interaction concept, we demonstrate that the physician in the operating room can interact intuitively with the platform. The dashboard also enables its users to gain access to external sources such as the HIS and start the required apps during surgery. We introduced 4 proof-of-concept apps to demonstrate how new software solutions can be easily integrated into the platform. The entire OP 4.1 prototype was tested with the medical use case *kidney tumor*. The aim of the dashboard is to provide the physician with the appropriate information at the right time (Figure 4). The required patient information, including information from a connected HIS, can be requested using natural language through built-in speech recognition or by manual input through a touch screen. Apps can be started centrally by a physician or a nurse through the dashboard. By sending push notifications to mobile devices through the *mobile information service* app, relevant stakeholders such as the surgery team are informed about the start or delay of planned operations, independent of their location. This is based on the app’s ability to retrieve current intraoperative time stamps from the cloud platform through APIs. By combining images of computed tomography scans from connected systems through the central cloud platform with live ultrasound images, the *precision puncture* app (Figure 5) enables a safe and fast percutaneous puncture of target structures, for example, in the kidney. The *augmented reality* app can display risk structures intraoperatively based on the segmentation of computed tomography images and the overlay of a 3D view of the kidney tumor in the laparoscopic video stream. The *live perfusion measurement* app enables the continuous quantification of renal tissue oxygenation with multispectral image analysis and machine learning. This allows the physician in partial kidney resection to determine which part of the kidney is still perfused after selective arterial clamping, helping to reduce the risk of kidney injury compared with hilar clamping [37].

The functionality of the OP 4.1 apps and data streams was demonstrated in real time at the conclusion of the project OP 4.1.

### The OP 4.1 Platform Business Model Concept to Create Tangible Value

Besides the development of a technology platform, we created a concept for a platform business model. To be commercially viable, the OP 4.1 platform approach needs to create value for all parties involved by facilitating exchanges among several independent market contenders: on the platform, consumers and providers interact with each other, with providers interacting among themselves as well, exchanging value (Figure 1). Software developers provide apps and digital content, and device manufacturers provide access to medical devices and related information in real time. Essential for a platform business model is a constant revenue stream for all market contenders as well as transparent pricing. Here, hospitals pay software developers and device manufacturers according to their respective agreed-upon payment model, software developers consuming device services pay device manufacturers for consuming these services, and software developers charge device manufacturers for software solutions such as predictive maintenance.

**Figure 6:**
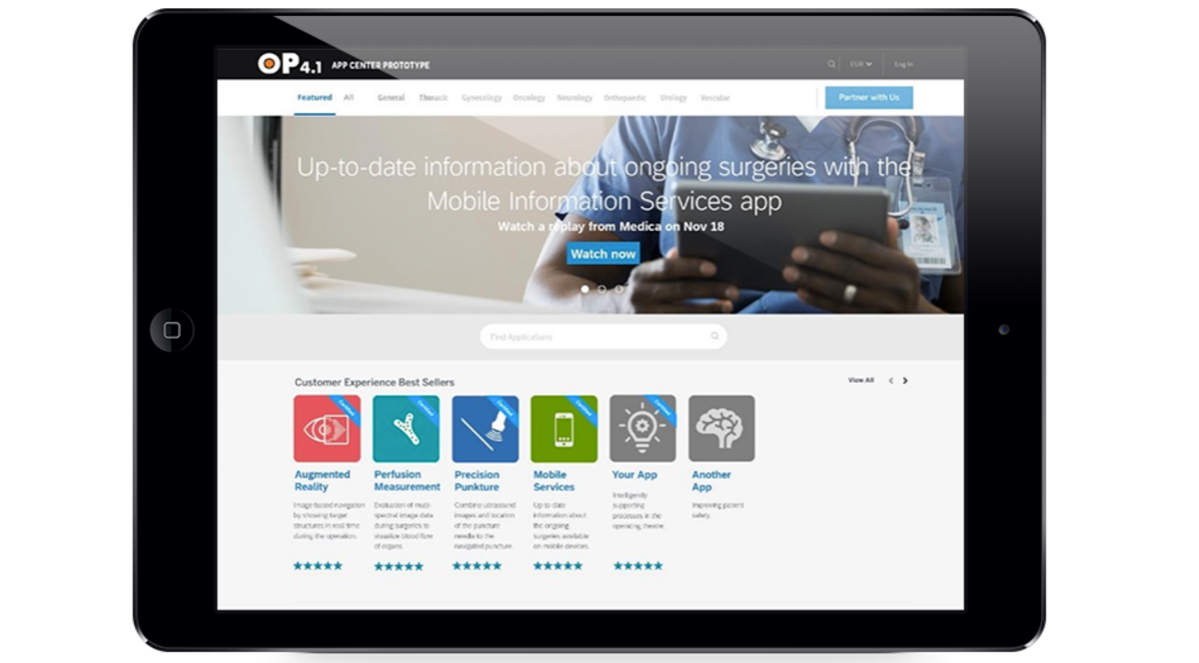
OP 4.1 app center. At the app center, users such as physicians and nurses could preselect apps for a specific surgery based on their filter criteria, recommendation, and previous social feedback from other users.

To access the platform, various channels and interfaces such as mobile apps, websites, digital stores, web services, or physical devices can be envisioned. Being the central entity, the platform provides interfaces to consumers, providers, and platform developers: consumers select and test apps on the OP 4.1 app center (Figure 6), providers expose and publish their microservices in the OP 4.1 API Hub as well as upload their apps, and platform developers can extend the platform through new standard services or additional medical gateways. Eventually, the platform will provide rule-based quality assurance of interfaces and certification for technical compliance, machine learning–based quality assurance, and social-based quality assurance through the ratings on the OP 4.1 app center as well as consumer feedback.

It is worth noting that the OP 4.1 platform prototype is a cooperation project among small and large German companies and research institutions, with an open and extensible approach. During the project, other companies (eg, Drägerwerk AG & Co. KGaA, Intuitive Surgical, Inc., and RaySearch Laboratories AB) expressed their interest in the OP 4.1 prototype and became associated partners. Such new partners could expand and enrich the platform with additional functionality.

### The Pay-Per-Use Model to Effectively Capture Value

**Figure 7:**
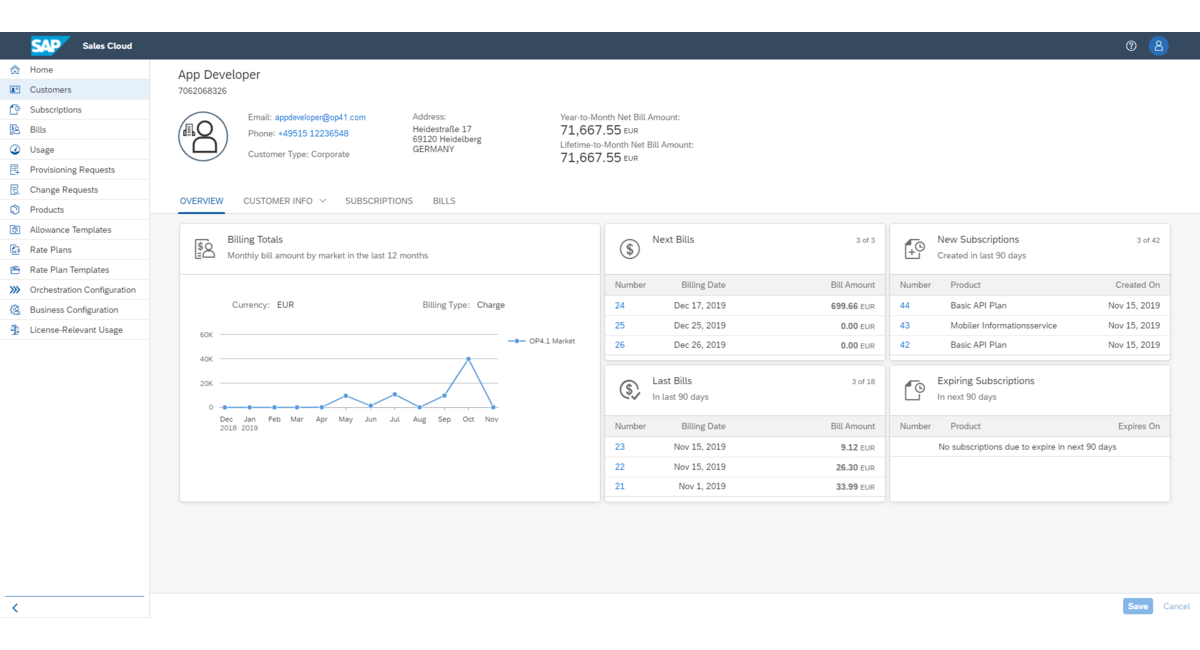
OP 4.1 commercial dashboard. On a single dashboard, the providers on the OP 4.1 platform can review and manage the commercial side of their activities. Among many other features, they can drill down into their customer base, see an overview of paid subscriptions, review bills and usage, and manage their products and commercial plans.

To help research institutes to monetize their inventions and intellectual property, we designed a flexible pay-per-use model as part of the OP 4.1 platform business model. Currently, medical devices are usually financed through a one-time payment of the product price as capital expenses plus recurring service and support fees as operational expenses. This makes the availability of highly innovative new solutions in the health care sector difficult because of limitations in the availability of capital. A business platform such as the OP 4.1 prototype can help to fill a market gap by supporting innovative payment terms for medical devices and software-based innovation. Information about device activity and specific modes will be transmitted to the integration platform. The platform can automatically provide the use records of devices and apps, thus enabling direct time-based or use case–based billing (Figure 7).

**Figure 8:**
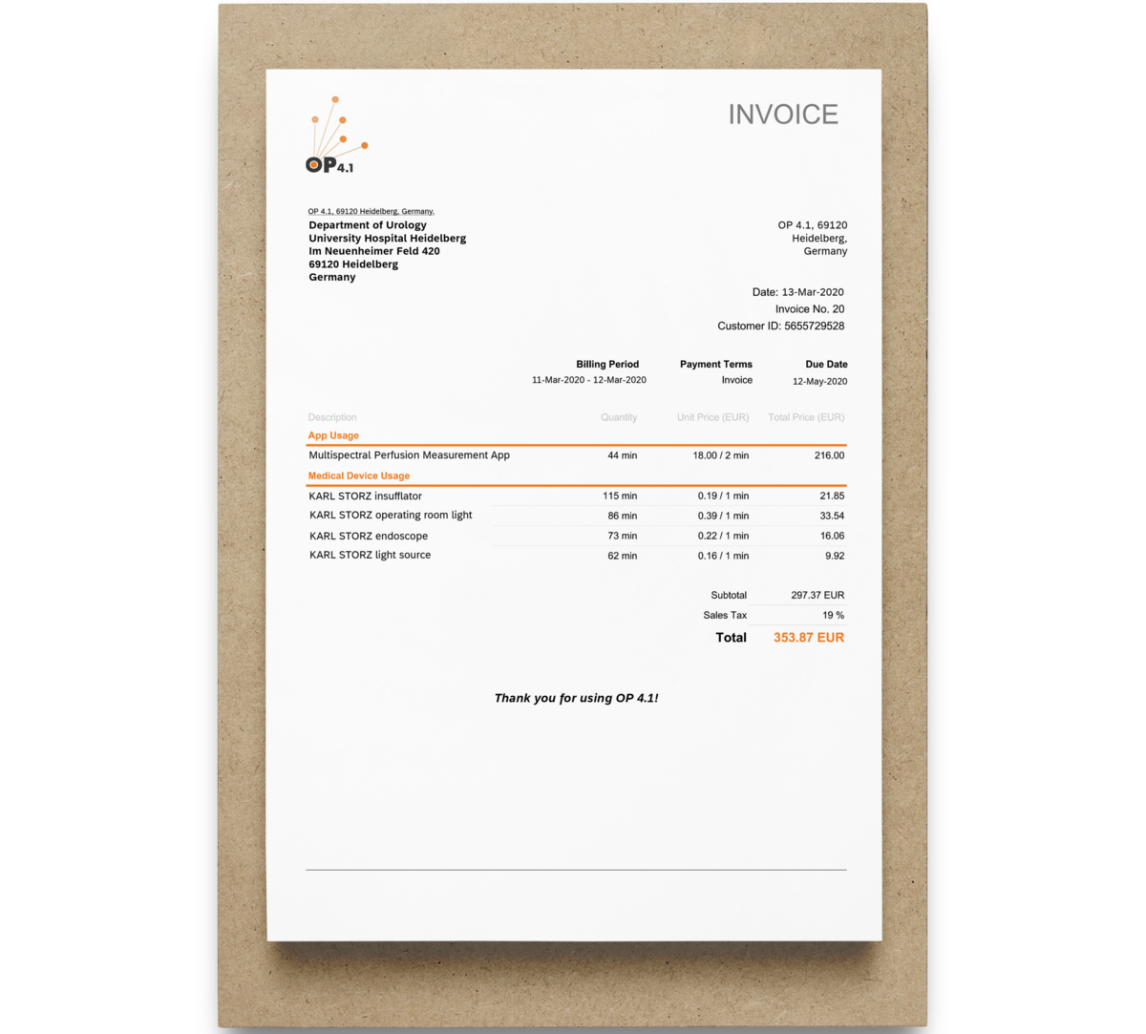
OP 4.1 platform invoice and pay-per-use model. The invoice generated by the OP 4.1 platform consolidates all resources used for a specific procedure, for example, medical devices, apps, and expendables, and charges for them in a single invoice according to their respective underlying commercial models.

We proved how to generate a consolidated invoice for the OP 4.1 showcase as shown in Figure 8. Such a service-oriented, pay-per-use model would have the advantage of not only spreading high, one-time investment costs over time, but it would also allow converting of capital expenses for highly innovative and beneficial equipment into operational expenses by allocating the exact costs during the intervention on a patient-related basis.

Currently, there is a strong trend from product sales through selling services to selling outcomes, which materializes in seven atomic business models across industries: classical, physical price models such as (1) give away device for free *(gift)* and (2) pay per device *(buy physical part)* as well as use-bound pricing models such as (3) pay per time that device is owned *(rent)*, (4) pay per time that device is used *(pay per time used)*, (5) pay per capability that device has delivered *(pay per [micro]service provisioned)*, (6) pay per number of cases that device contributed to *(pay per incident)*, and (7) pay per total outcome of device’s use *(pay by outcome).* For the OP 4.1 platform, it can be envisioned to offer pricing models based on transaction fees (eg, pay per activity or use), subscription fees (eg, pay per time), and lead-generation fees (eg, pay by transaction initiated or facilitated). Building on this concept, it can be foreseen that all potential device use scenarios eventually map to a combination of these pricing models, enabling even more exotic pricing models on the platform to support the varying use cases encountered in real-world hospital scenarios.

## Discussion

### Principal Findings

The rapid translation of software-based medical research and its results into clinical routine to improve patient outcomes is confronted with structural hurdles, even in the era of Industry 4.0. Innovative solutions are affected by difficult access to medical device data and high barriers to market entry because of proprietary systems. In this proof-of-concept project, we demonstrate, to the best of our knowledge for the first time, how these issues can be addressed by a technology and business platform for the operating room. Through the integration of different perioperative process data and the connection of clinical systems with patient data, it offers a basis for the development and implementation of new and innovative solutions in the form of apps. In the project OP 4.1, we successfully developed a cloud-based integration platform that also provides a business model and related platform. This business platform not only enables fast access to published device data and relevant patient data, but also allows charging for apps or devices, that is, through pay per use. Thus, we effectively created a new market for purely software-based solutions, helping to transfer technical innovation into clinical routine and to become commercially successful. We anticipate that with the distribution of smart, software-based medical solutions through the platform the treatment of patients can become safer and more precise.

The OP 4.1 platform could create benefit for *public health*, *physicians* and *hospitals*, *device manufacturers*, *software developers*, and ultimately *patients*. With the percentage of people aged ≥65 years doubling until 2050 and with an increasing population in general [38,39], *public health* is confronted with demands to be both productive and progressive. Medicine is facing large numbers of newly developed medical devices that need to interact with each other. Whereas other industries have already embraced IoT, in health care the Internet of Medical Things [40] is still in its infancy in terms of supporting the treatment of patients. Further digitalization of the health care sector can help to create a medical environment that is more predictive, preventive, personalized, and participatory [41].

At *hospitals*, *physicians* are the primary active users of platforms such as OP 4.1, leveraging apps offered through app centers. The expansion of software-defined healthcare helps to strengthen navigated, smartly assisted procedures, allowing *physicians* to take better and quicker decisions. *Physicians* benefit from quality assurance in real time (eg, live perfusion measurements) and potential procedural improvements (eg, improving the outcome of a surgery with augmented reality apps). We also anticipate that platforms could help physicians to increase productivity (eg, transfer from surgery data into surgery protocol), leveraging an existing HIS connected to the platform. In addition, through the transparent logging of process data, such platforms facilitate quality control for *hospitals* and the implementation of new models for commercialization of apps and devices through pay per use, allowing the conversion of investment costs into intervention-related operational costs. The business model is attractive to hospitals not only for being able to shift cost types such as capital expenses to operational expenses, but also for being able to allocate expenses that are normally considered overheads to individual patient treatments, subject to local regulations. The business model also allows hospitals to charge device manufacturers and software developers for evaluating new products and for providing user feedback. In addition, apps allow hospitals to obtain further benefits through more efficient processes and cost savings. The more efficient use of technology, information, and personnel in interventions can contribute to long-term cost reduction as well as optimization of work processes at *hospitals*.

Established *device manufacturers* can use platforms such as the OP 4.1 prototype to create and offer an ecosystem of connected solutions and even vendor-brand them. Traditionally, products sold by *device manufacturers* to hospitals represent optimized but self-contained stand-alone solutions. The usage data provided by the platform enables *device manufacturers* to continuously improve and adapt their solutions based on the needs of users that are derived from the information available in the operating room. The provision of medical device data is important for data-driven applications, including resource management, process optimization, quality assessment, performance analysis, context-sensitive assistance, and predictive maintenance. This leads to the use of devices in new scenarios and faster and more effective innovation cycles.

For *software developers*, the successful integration of the 4 apps into the OP 4.1 prototype acts as a solid reference and proof of concept on how to implement apps on the platform and exemplifies the simplification of translation of innovation into clinical routine. The 4 apps developed as part of the OP 4.1 prototype act as templates for designing and coding for other software developers. As functionality is replicated across the platform, once developers know how to create a module, further development can be performed more quickly. In addition, the platform provides central services to developers for developing and exposing their solutions to the customers through an app center. *Software developers* creating new software solutions get access to other providers’ device simulations through the platform and can augment device capabilities by applying new software solutions. Eventually, *software developers* will potentially require less time and effort to deliver new solutions (eg, through predefined design concepts). Ultimately, translational research departments benefit from a quick and cost-effective transfer of their innovative results to multiple customers. The platform prototype gives researchers access to a large customer market (eg, hospitals that already participate in such platforms). This avoids a scenario where only *patients* of selected hospitals benefit from the latest innovation while the respective clinical trial is open and active. Cultural and socioeconomic health care systems vary widely across Europe and worldwide, with many systems approaching health care as a business, sometimes at the risk of implementing 2-tier or multitier medicine. New models for commercialization of apps and devices through pay per use do not work in this dimension, but they offer commercial benefits such as allowing the conversion of investment costs into intervention-related operational costs. In new business models, all *patients* could benefit from innovation, even if invoicing is just occurring for *patients* with, for example, private insurance. Software-defined healthcare can even be cheaper for hospitals because it is often not necessary to buy new equipment; instead, the functionality of the existing equipment can be extended by means of software upgrades or add-ons.

Thus, the OP 4.1 platform can lower the barrier to market entry and efficiently make innovations available to all platform participants as well as patients.

### Comparison With Previous Work

New opportunities for data collection have been created by the ubiquitous availability of mobile devices and wearables. Advances in health platforms (eg, Apple HealthKit and Google Fit) allow the bundling of fitness and medical data from different sources and make these available for sharing with health care professionals [42]. The aim of the SMART project was to develop an open platform to enable medical apps to run unmodified across different health care IT systems, promoting interoperable and vendor-independent apps [43]. The Medical Device Plug-and-Play Interoperability Program has been promoting medical device interoperability to enable the creation of complete electronic health records and cost-effective development of medical apps when using networked medical devices in clinical routine [44]. Another example is OR.NET, which defined cross-manufacturer concepts for the dynamic and secure networking of medical devices and IT systems [23]. Further research was performed in the project InnOPlan, which developed a SmartData platform for the real-time provision and analysis of medical device data to enable data-driven services in the operating room [45]. The HiGHmed Consortium aims at establishing data integration centers and an open platform architecture in cooperation with health care providers in the fields of oncology, cardiology, and infection control so that integration and reuse of data are facilitated [46]. The HiGHmed Consortium is one of the members of *the German* *Medical Informatics Initiative*, whose goal is to advance digitization in health care research and the exchanging of patient data, specifically among university hospitals. Data integration centers will enable research data to be collected and integrated across several institutions and locations [47].

Our approach goes far beyond previous efforts that focus primarily on data integration in medicine. With the OP 4.1 platform, we demonstrated for the first time a cloud-based integration platform that also provides a business model and related platform, thus effectively creating a new market for purely software-based solutions and helping to transfer technical innovation into the operation room and clinical routine and to become commercially successful.

### Future Directions

Going further, it is straightforward to envision several dimensions in which to expand our findings in subsequent projects: big *data* feeding AI algorithms, the *expansion* of stakeholders on the platform, and the *transfer* of the platform concept into other medical disciplines.

The available *data* in the operating room can be permeated through the integration of devices from various companies, with the OP 4.1 platform enabling the development of solutions that span >1 device type. By introducing a consistent system to capture information across the operating room, it will be possible to create solutions analyzing current situations and patients’ states as well as simulating outcomes, consequently predicting future states and proposing next steps for the interventions. High-quality data sets dynamically generated on a per-patient basis through medical devices and central clinical *data* collection are ideal for machine learning algorithm training [48,49]. Big data analysis will play a significant role in transforming medicine, and technology that enables the central organization, processing, and security of these data is critical [50-52]. The integrative and expandable OP 4.1 platform concept can provide the registration and analysis of diverse results from diagnostics and therapy in real time and over time. As a result, a data infrastructure could be created, supporting the next major step into data-based individualized medicine with its personalized and customized therapies [53].

The *expansion* of a platform such as OP 4.1 could attract many companies, thus starting self-supporting network effects that lead to higher numbers of devices being made available on the platform and more developers developing on the platform, resulting in more solutions available on the platform, and finally more consumers using these solutions and hospitals subscribing to the platform. In the long term, an ecosystem of producers and consumers, including various hospitals, device manufacturers, and software developers, can be curated on the platform.

In addition, the concept of the OP 4.1 prototype is not meant to be restricted to an operating room; a *transfer* to other medical disciplines is a viable option. Various disciplines could benefit, for example, through less effort having to be made for point-to-point integration, quality control, as well as the simple integration of systems by different vendors. The use of the OP 4.1 platform in multiple departments of a hospital enables central patient data management and analysis across the existing clinical information systems, exploiting potential synergies.

A business platform such as the OP 4.1 platform prototype can support several rising industry trends such as the transformational trend *digital health platform* for hospitals. The concept *digital health platform* as the architecture enabling the composable enterprise for health care providers supports the evolution from electronic medical records to a *hospital without walls* by enabling these organizations to rapidly adapt to changing patient demand, partner capabilities, and industry trends. This concept can be realized by leveraging packaged business capabilities and is anticipated to become mainstream in 2025-2030. Further major industry directions are health data curation and enrichment, AI-enabled diagnostic imaging interpretation, and particularly an app marketplace for health care providers [54]. All these are included or could easily be enabled by a platform such as OP 4.1. Natural extensions of the OP 4.1 platform could focus on digital twins in health care, precision health, and medicine, as well as AI health care advisors. Furthermore, there is a strong potential to not only promote IoT in health care, a growing area of research and another transformational trend with the opportunity for health care systems to predict health issues and to monitor patients in various institutions [55], but to also help to make it commercially viable through the concept of a multisided market that we introduced with OP 4.1.

### Limitations

This study includes certain limitations. In the proof-of-concept project OP 4.1, we developed a prototype. During the development of the OP 4.1 platform for implementation at different institutions, further challenges and tasks will arise. The OP 4.1 platform prototype would need to be adapted to hospital-specific infrastructures and requirements; therefore, it would need to be delivered as a customer-specific solution.

In the OP 4.1 project, 4 apps in the field of urology for the medical use case *kidney tumor* were developed. These apps demonstrated as a proof of concept the feasibility of developing and integrating apps into the OP 4.1 platform prototype. The verification of the superiority of these apps compared with standard procedures, that is, through validation with real-world data, is pending. In this proof-of-concept project, we did not present quantitative data regarding an actual implementation on the OP 4.1 platform. After having proven the functionality of integrating medical research as apps into the OP 4.1 platform, the next step will be to prospectively validate the individual apps in clinical routine according to their cost-benefit ratio, patient safety, and improved clinical outcomes.

Going forward, to expand the scope for other medical disciplines, more apps need to be integrated into the OP 4.1 platform. The number of valuable apps needs to be expanded and adapted to the different medical disciplines and customer needs.

It should also be noted that before apps can be released for distribution through a platform such as OP 4.1, they would need to be certified and approved for use. It is important to ensure that the respective rules of medical device regulations are strictly observed. Personal data security and protection compliance as well as medical device certifications need to be fulfilled by each software developer individually because only they know which data are generated, stored, processed, and so on.

### Conclusions

In the proof-of-concept project OP 4.1, the prototype of a user-centric, open, and extensible platform for the intelligent support of processes in the operating room was developed. By connecting data sources and medical devices from different manufacturers, the technology and business platform creates a multisided market for the successful development, implementation, and accounting of innovative software solutions in health care. Consequently, software-based medical innovation can be translated into clinical routine quickly, efficiently, and cost-effectively, optimizing the treatment of patients through smartly assisted procedures.

## Abbreviations

AI: artificial intelligence
API: application programming interface
DKFZ: Deutsches Krebsforschungszentrum (German Cancer Research Center)
HIS: hospital information system
IoT: internet of things
IT: information technology
